# *Anaplasma bovis*–Like Infections in Humans, United States, 2015–2017

**DOI:** 10.3201/eid2909.230559

**Published:** 2023-09

**Authors:** Sandor E. Karpathy, Luke Kingry, Bobbi S. Pritt, Jonathan C. Berry, Neil B. Chilton, Shaun J. Dergousoff, Roberto Cortinas, Sarah W. Sheldon, Stephanie Oatman, Melissa Anacker, Jeannine Petersen, Christopher D. Paddock

**Affiliations:** Centers for Disease Control and Prevention, Atlanta, Georgia, USA (S.E. Karpathy, C.D. Paddock);; Centers for Disease Control and Prevention, Fort Collins, Colorado, USA (L. Kingry, S.W. Sheldon, S. Oatman, J. Petersen);; Mayo Clinic, Rochester, Minnesota, USA (B.S. Pritt, J.C. Berry);; University of Saskatchewan, Saskatoon, Saskatchewan, Canada (N.B. Chilton, S.J. Dergousoff);; University of Nebraska, Lincoln, Nebraska, USA (R. Cortinas);; Minnesota Department of Health, St. Paul, Minnesota, USA (M. Anacker)

**Keywords:** *Anaplasma bovis*, human anaplasmosis, *Dermacentor variabilis*, *Dermacentor andersoni*, zoonoses, vector-borne infections, United States, bacteria

## Abstract

We detected the DNA of an *Anaplasma bovis*–like bacterium in blood specimens from 4 patients from the United States with suspected tickborne illnesses. Initial molecular characterization of this novel agent reveals identity to *A. bovis*–like bacteria detected in *Dermacentor variabilis* ticks collected from multiple US states.

The genus *Anaplasma* includes several species of tickborne, zoonotic pathogens of global importance. Three recognized species (*Anaplasma phagocytophilum*, *Anaplasma ovis*, and *Anaplasma bovis*) and one provisionally named species (*Anaplasma capra*) are associated with moderately severe to severe disease in humans (*1*). Human infections with *A. bovis*, a pathogen first identified in monocytes of cattle in Algeria in 1936 and subsequently detected in other countries in Africa, Asia, and the Americas, were reported from China in 2017 (*1*–*3*). In 2015, a targeted metagenomic approach designed to amplify the V1–V2 region of the bacterial 16S rRNA (*rrs*) gene identified DNA of an *A. bovis*–like agent in blood specimens from 2 US patients with suspected tickborne illnesses (*4*). The agent demonstrated 100% identity across a 357-bp region of *rrs* to *A. bovis*–like sequences amplified from several human-biting *Dermacentor* tick species in North America (*4*). An additional 2 US patients positive for this same *Anaplasma* species were identified in 2017 (L. Kingry et al., unpub. data), although the genetic identity of this pathogen remained limited to the same 357-bp sequence of *rrs* (*5*–*7*). To further characterize the phylogenetic position of this novel agent, we evaluated additional sequences to determine the uniqueness of this strain among the expanding global complex of *A. bovis*–like bacteria.

## The Study

We extracted DNA from 100 µL of EDTA-treated whole blood obtained from 4 patients from whom partial *rrs* sequences of an *A. bovis*–like agent were identified from a targeted metagenomics assessment of whole blood specimens collected from US patients with suspected tickborne disease (*4*; L. Kingry et al., unpub. data). DNA extracts containing *A. bovis* DNA were also available from an adult *Dermacentor andersoni* tick collected in Saskatchewan Landing Provincial Park in Saskatchewan, Canada, and from 5 adult *Dermacentor variabilis* ticks collected in Washita County, Oklahoma; Floyd County, Iowa; and Sarpy and Cass Counties, Nebraska, from which partial *rrs* sequences most similar with *A. bovis* were amplified previously (*5*,*6*; L. Kingry).

We amplified segments of the *rrs*, citrate synthase (*glt*A), and heat shock chaperon (*gro*EL) genes using Taq PCR Master Mix Kit (QIAGEN, https://www.qiagen.com) (Table 1). Each 20-µL primary reaction consisted of 1 µM of each primer, 10 µL Taq Master Mix, 2 µL DNA, and 6 µL molecular-grade water. Secondary reactions (*gro*EL only) consisted of 1 µM of each primer, 10 µL Taq Master Mix, 1 µL primary PCR product, and 7 µL molecular-grade water. We resolved PCR amplicons on a 1% agarose gel in Tris-acetate-EDTA buffer and cut amplicons from the gel and purified using a Wizard SV Gel and PCR Clean-up kit (Promega, https://www.promega.com). We sequenced each purified amplicon (1 µL) bidirectionally using a Big Dye Terminator v3.1 Cycle Sequencing Kit, purified using a BigDye XTerminator Purification Kit, and sequenced using an ABI 3500 Genetic Analyzer (all from ThermoFisher Scientific, https://www.thermofisher.com).

**Table 1:** PCR primers used in study of *Anaplasma bovis*–like infections in humans, United States, 2015–2017

Gene	Primer name	Sequence, 5′ → 3′	Annealing temperature*	Reference
*rrs*	Out2F	GAT AGC GGA ATT CCT AGT GTA GAG GTG	56°C	(*8*)
	317Pan	AAA GGA GGT AAT CCA GC		
*glt*A	Abov_gltA2F	CGG AAA TTA CTT TTA TAG ATG G	49°C	This study
	Abov_gltA2R	CAT ACC AYT GAG AAA CCC AAC		
gro*EL*	HS1-f	CGT CAG TGG GCT GGT AAT GAA	54°C	(*9**,**10*)
	HS6-r	CCW CCW GGT CWA CAC CTT C	50°C	(*11*)
	HS3-f	ATA GTY ATG AAG GAG AGT GAT		
	HSVR	TCA ACA GCA GCT CTA GTW G		

*Cycling conditions: 95°C for 3 min followed by 35 cycles of 95°C for 30 s, 1 min at the annealing temperature listed above, and an extension at 72°C for 1 min 30 s. This was followed by a final extension at 72°C for 10 min.

We used Geneious Prime version 2021.0.3 (https://www.geneious.com) to assemble and align consensus sequences and infer the phylogenetic relationships between DNA sequences (*12*). Only 3 sources of genetic information for *A. bovis* were available in GenBank that provided complete or partial sequence data at all 3 loci, including those amplified from the blood of a raccoon (*Procyon lotor*) captured in Hokkaido, Japan (*13*); a goat (*Capra* sp.) from Shaanxi Province, China; and a cow (*Bos taurus*) from Shaanxi Province, China. The *rrs, glt*A, and *gro*EL nucleotide sequences amplified from the human samples were submitted to GenBank and assigned the accession numbers OQ693620 (*rrs*), OQ694770 (*glt*A), and OQ693619 (*gro*EL).

The *rrs* sequences (599-bp) of the 4 human samples were 100% identical to each other and to those amplified from a *D. andersoni* tick and 5 *D. variabilis* ticks; the sequences also showed 98.3% identity to the *rrs* sequences amplified from blood specimens obtained from the cow from China, 98% to those from the goat from China, and 97.8% identity to those from the raccoon from Japan. The 826-bp *glt*A sequences from the 4 human samples were 100% identical to each other and to all sequences from *D. variabilis* ticks; they also were 99.4% identical to the 827-bp sequence from the *D. andersoni* tick*.* When trimmed to 356 bp to match the sequence lengths available in GenBank of those from the cow and goat from China, the North America sequences amplified from humans and ticks shared only 78.6%–79.4% identity with the sequences from China. The *gro*EL sequences (1,079-bp) of the human samples were 100% identical to each other and to the corresponding sequences amplified from all 5 *D. variabilis* ticks and showed 99.4% identity to the *gro*EL sequence amplified from the *D. andersoni* tick. Those samples showed only 85.4% identity to the *A. bovis* sequences from the raccoon from Japan and 84.6% identity to the sequences from the cow and goat from China. Phylogenetic analyses using concatenated sequences from the 3 loci produced an inferred consensus tree that grouped human and North America *Dermacentor* spp. tick samples with the other *A. bovis* sequences but with strong statistical support (100%) for the separation of *A. bovis*–like sequences from North America and those from China and Japan (Figure).

**Figure:**
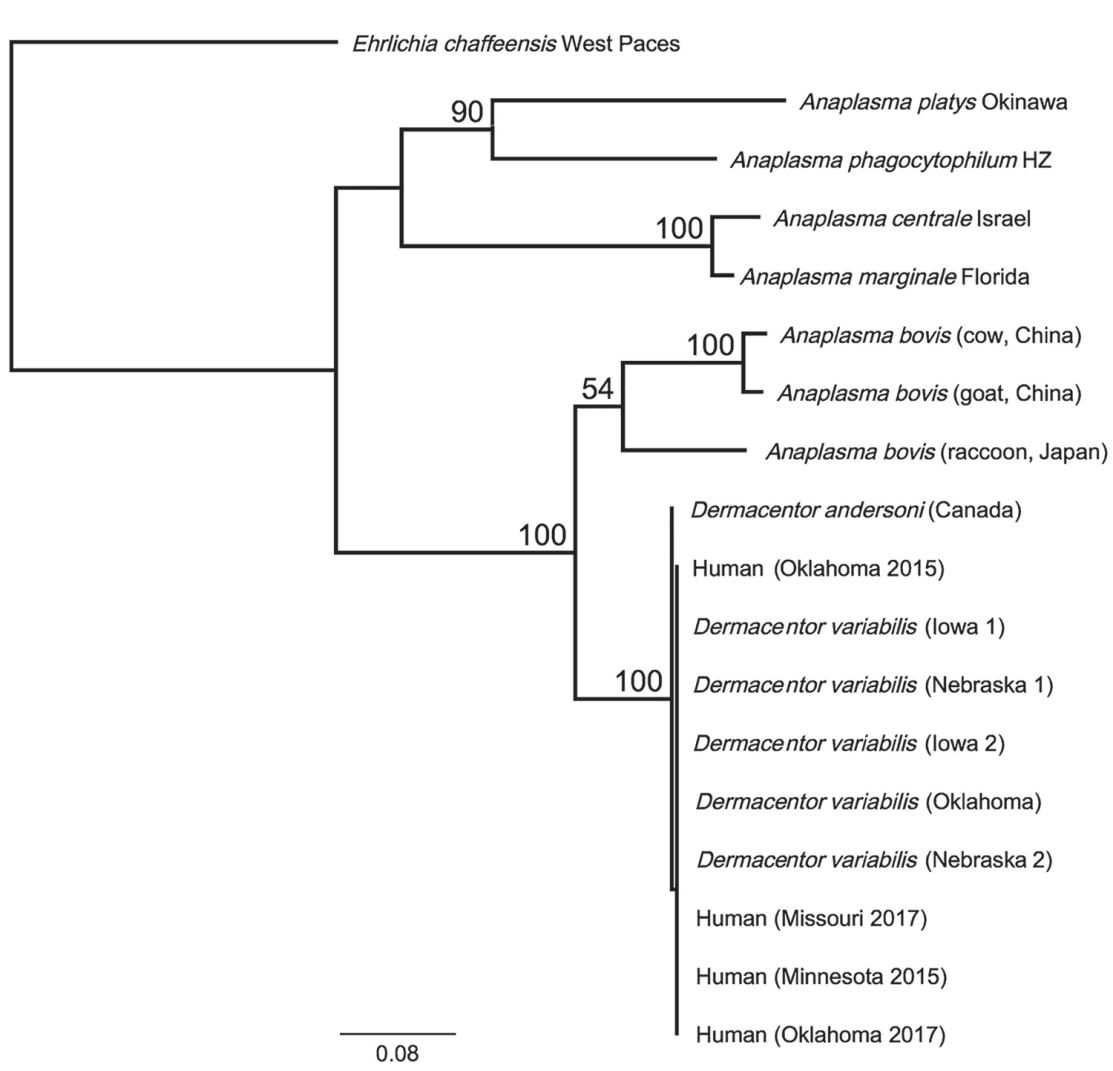
Phylogenetic relationship of novel human *Anaplasma bovis*–like pathogen associated with human cases in the United States, 2015–2017, to other *A. bovis*–like and related *Anaplasma* species based on 2,039 bp of concatenated *rrs*, *glt*A, *gro*EL nucleotide sequences. Phylogenetic relationships were inferred using the RAxML method using the general time reversible plus gamma model (*13*). One thousand bootstrap replicates were used to estimate the likelihood of the tree; bootstrap values are displayed next to the nodes. Only bootstrap values of >50 are shown. GenBank accession numbers for the samples in this study: OQ772254;, *glt*A; OQ772255, *gro*EL; and OQ724830, *rrs*; those for the *D. andersoni* sample were assigned the following numbers: OQ772256, *glt*A; OQ772257, *gro*EL; and OQ724821, *rrs.* Reference sequences from GenBank: *Anaplasma bovis* (cow, China): MH255937, 16S; MH594290, *glt*A; MH255906.1, *gro*EL; *A. bovis* (goat, China): MH255939, 16S; MH255915.1, *glt*A; MH255907, *gro*EL; *A. bovis* (raccoon, Japan): GU937020, 16S; JN588561, *glt*A; JN588562, *gro*EL; *Anaplasma platys* strain Okinawa: AY077619, 16S; AY077620, *glt*A; AY077621, *gro*EL; *A. phagocytophilum* strain HZ NC_007797; *A. centrale* strain Israel NC_013532; *A. marginale* strain Florida NC_012026. *Ehrlichia chaffeensis* strain West Paces (NZ_CP007480) was used as the outgroup. Scale bar represents mean number of nucleotide substitutions per site.

## Conclusions

A novel and presumably tickborne pathogen of humans was identified in blood of patients from the central and upper midwestern United States during 2015–2017 (Table 2). The amplification of a thus far genetically identical agent from *D. variabilis* ticks suggests that this tick species could represent a vector of this *A. bovis*–like agent in the United States. This bacterium is also related to a worldwide complex of bacteria, detected in multiple species of ticks and domesticated and wild animals, designated collectively as *A. bovis*. Because *A. bovis* has never been cultured in vitro, neither a type strain nor a complete genome exist for this pathogen. Only 3 genetic loci from *A. bovis* exist in GenBank, and few sources provide complete sequences for all loci from the same sample. As seen in this evaluation, the level of nucleotide identity among samples can vary considerably at an individual locus and hamper efforts to establish genetic relatedness of *A. bovis*–like bacteria.

**Table 2:** Demographic and geographic characteristics associated with human cases of *Anaplasma bovis*–like infection identified in the United States, 2015–2017

Specimen	Patient age, y/sex	State of origin	Date of collection
Oklahoma 2015	71/F	Oklahoma	2015 Jun 9
Minnesota 2015	34/M	Minnesota	2015 Aug 12
Oklahoma 2017	67/F	Oklahoma	2017 May 21
Missouri 2017	54/M	Missouri	2017 Jun 14

The spectrum of disease and epidemiology associated with human infections caused by this novel *A. bovis*–like agent remains unknown. Presumably, human infections with this agent in the United States are uncommon, because this bacterium was detected only 4 times from 29,928 residual clinical samples obtained during 2014–2019. By comparison, 1,236 infections with *A. phagocytophilum* and 345 infections with *Ehrlichia* spp. were identified from this investigation during the same period (*5*; L. Kingry et al., unpub. data). The study design that enabled the discovery of this novel agent also precluded the collection of clinical details of infected patients; nonetheless, an *A. bovis*–like pathogen was detected recently in blood of patients from Anhui and Jiangxi Provinces in China who had illnesses characterized predominantly by fever, myalgia, fatigue, anorexia, and thrombocytopenia (*3*). In the United States, *A. bovis*–like bacteria have been detected in blood samples from cottontail rabbits (*Sylvilagus* spp.) from Massachusetts, Georgia, and Texas and from black-tailed jackrabbits (*Lepus californicus*) from Texas (*14*,*15*). Developing a specific molecular assay could help identify additional patients infected with this novel agent and clarify the tick and wildlife species involved in its natural history and transmission to humans.
